# Benchmarking alcohol policy based on stringency and impact: The International Alcohol Control (IAC) policy index

**DOI:** 10.1371/journal.pgph.0000109

**Published:** 2022-04-22

**Authors:** Sally Casswell, Taisia Huckle, Karl Parker, Jose Romeo, Thomas Graydon-Guy, June Leung, Karimu Byron, Sarah Callinan, Surasak Chaiyasong, Ross Gordon, Anne Marie MacKintosh, Petra Meier, Guillermo Paraje, Charles D. Parry, Cuong Pham, Petal Petersen Williams, Steve Randerson, Karen Schelleman-Offermans, Gantuya Sengee, Perihan Torun, Wim van Dalen, Nadine Harker

**Affiliations:** 1 SHORE & Whariki Research Centre, College of Health, Massey University, Auckland, New Zealand; 2 National Council on Drug Abuse Prevention, Basseterre, St Kitts and Nevis; 3 Centre for Alcohol Policy Research (CAPR), School of Psychology and Public Health, La Trobe University, Melbourne, Victoria, Australia; 4 International Health Policy Program (IHPP), Ministry of Public Health, Nonthaburi, Thailand; 5 Faculty of Health Sciences and Sport, Institute for Social Marketing and Health, University of Stirling, Stirling, Scotland, United Kingdom; 6 School of Health and Related Research, University of Sheffield, Sheffield, England, United Kingdom; 7 Business School, Universidad Adolfo Ibáñez, Santiago, Chile; 8 Alcohol, Tobacco and Other Drug Research Unit, South African Medical Research Council, Cape Town, Western Cape, South Africa; 9 Center for Injury Policy and Prevention Research (CIPPR), Hanoi University of Public Health, Hanoi, Vietnam; 10 Faculty of Psychology & Neuroscience, Work & Social Psychology, Maastricht University, Maastricht, The Netherlands; 11 Public Health Policy and Coordination Department, National Center for Public Health of Mongolia, Ulaanbaatar, Mongolia; 12 Department of Public Health, Hamidiye International Medical School, Istanbul, Turkey; 13 Dutch Institute for Alcohol Policy STAP, Utrecht, The Netherlands; South African Medical Research Council, SOUTH AFRICA

## Abstract

This study developed a measurement tool to assess stringency and ‘on-the-ground’ impact of four key alcohol policy domains to create an alcohol policy index suitable for benchmarking alcohol policy and assessing change over time in middle- and high-income countries. It involved a collaboration between researchers in 12 diverse countries: New Zealand; Australia; England; Scotland; Netherlands; Vietnam; Thailand; South Africa; Turkey; Chile; Saint Kitts and Nevis and Mongolia. Data on the four most effective alcohol policy domains (availability, pricing policy, alcohol marketing, drink driving) were used to create an alcohol policy index based on their association with alcohol per capita consumption (APC) of commercial (recorded) alcohol. An innovation was the inclusion of measures of impact along with the stringency of the legislation or regulation. The resulting International Alcohol Control (IAC) Policy Index showed a very high negative correlation (-0.91) with recorded APC. Greater affordability of alcohol, an impact measure taking into account prices paid and countries’ Gross Domestic Product, was predictive of higher APC (-0.80). Countries in which more modes of alcohol marketing are legally allowed and used had higher APC. Legislation on outlet density and drink driving predicted APC whereas trading hours did not. While stringency and impact measures varied between domains in terms of relationship with APC, overall, there was a strong correlation between impact and stringency (0.77). The IAC Policy Index, which includes measures of policy stringency and ‘on-the-ground’ impacts in relation to four key policy areas, was found to be strongly associated with commercial alcohol consumed in a number of diverse country settings. It showed a larger relationship than previous indices that include more policy dimensions. The index provides a relatively simple tool for benchmarking and communication with policy makers to encourage a strong focus on uptake of these four most effective alcohol policies.

## Introduction

### Global context

In 2018 alcohol attributable deaths amounted to three million globally per annum. This burden is expected to rise due to increased consumption in low- and middle-income countries (LMICs), especially in the South East Asia and Western Pacific regions, if effective policies requiring regulation of supply and marketing and increased taxation are not implemented [1].

Alcohol policies stand out among other non-communicable disease relevant policies for the lack of uptake [2]. In 2020 the Executive Board of the World Health Assembly, in response to concern expressed largely by LMICs [3], requested the Director General to develop an action plan for implementing the global strategy to reduce the harmful use of alcohol and to adequately resource work on the harmful use of alcohol [4]. This provides a new opportunity to increase the uptake of the effective policies, the ‘best buys’ identified by the UN [5], and highlights the need to include monitoring of this uptake at the country level [6].

### What gets measured gets done

Composite indicators are widely used by international organisations to compare country performance on issues ranging from health and development, to the economy and environment [7,8]. In the context of policy analysis, such indicators can be used by decision makers to benchmark policy development. Ultimately, a composite indicator should facilitate communication with relevant stakeholders, highlight where change needs to occur, and promote accountability by decision makers [7].

### Alcohol policy indices

A number of composite indicators have been developed to measure effects of alcohol control policies. Most studies have looked for a relationship linking indices with per capita alcohol consumption (APC) (available in the Global Information System on Alcohol and Heath (GISAH)). Cross-sectional analysis has generally found correlations [5,9], with stronger relationships reported in high-income countries (HICs) [10] compared with countries in Africa [11]. A study of countries from the WHO (World Health Organization) Western Pacific Region showed an association once APC was adjusted by gross domestic product (GDP) [12], and in Europe, with the exception of southern European countries, stricter policies were strongly associated with lower APC [13].

More recently, alcohol policy indices have been developed using policy data available in WHO’s Global Information System on Alcohol and Health (GISAH), covering larger and more diverse samples and including more dimensions of a country’s response to alcohol harm. One study that included the ten dimensions of the WHO global strategy found only a modest association between higher index scores and lower APC once the covariates GDP, population age, urbanisation, and world region were taken into account [14]. An index of alcohol policy in U.S. states was developed based on expert assessments of the implementation of 29 policies and reported relationship with alcohol harm [15–17].

The aim of the present study was to develop a policy index based on only a small number of the most effective and most relevant policies, and using data collected in-country with minimal resources, so therefore suitable for use in LMICs as well as high-income countries. An innovation in this study was to not only include data on the legislation pertaining to these policies (stringency), but also measures of the way in which these policies had actually affected key aspects of the alcohol environment (policy impact) using measures of the alcohol environment collected as part of the IAC Alcohol Environment Protocol [18].

This is the first stage of a study to develop and validate the IAC Policy Index based on the association with recorded APC. Data on recorded (commercially produced) alcohol was used based on the assumption that these policies are directed towards and will have a more direct effect on recorded alcohol consumption. This paper reports the first stage of this project, which was to develop and test the IAC Policy Index. The second stage is to apply the IAC Policy Index to cross-country survey data on specific drinking patterns–the results will be reported in future publications.

## Methods

### Participants

England, Scotland, Australia, New Zealand, South Africa, St Kitts and Nevis, Vietnam, Thailand, Chile, the Netherlands, Mongolia and Turkey were the jurisdictions included in the study [19]. This is a ‘convenience sample’ based upon those who countries who had obtained funding to participate in the IAC project. The sample is a small but heterogeneous group of countries. Data were collected for one year for each site for the period 2012 to 2020.

#### Policy domains

We examined regulatory domains identified by the WHO: three ‘best buys’ (restrictions on availability and marketing and pricing policies) and one ‘good buy’ (drink-driving prevention) as determined by effectiveness research [20–22] (Table 1). We did not include brief interventions (the other good buy) because our investigation focused on public health measures aimed at prevention. We included impact measures reflecting both policy stringency and implementation and enforcement. The domains reflected the policies most applicable to the general population and we excluded those directed specifically at younger people(e.g., minimum purchase age and social supply). Future work will develop a youth relevant policy index to better understand the impact of policies affecting only young people.

**Table 1:** Measures and weighting parameters used for the IAC policy index.

Policy status (legislated)	Effectiveness	Stringency of legislated policy		Impact on the ground
	Weight	Description	Score	
***Trading hours/days of sale***	***1–5***			
*On-premise*		Number of legal trading hours per day	0–1	Actual trading hours per day^2^
		Legally allowed to open 7 days	Yes 0/No 1^1^	Actually open 7 days
*Off-premise*		Number of legal trading hours per day	0–1	Actual trading hours per day
		Legally allowed to open 7 days	Yes 0 /No 1	Actually open 7 days
***Outlet density***	***1–5***			
*On-premise*		Restrictions on number	Yes 1 /No 0	Not included—not available for enough countries
		Restrictions within geographic area	Yes 1 /No 0	
		Restrictions from certain locations	Yes 1 /No 0	
*Off-premise*		Restrictions on number	Yes 1 /No 0	Not included—not available for enough countries
		Restrictions within geographic area	Yes 1 /No 0	
		Restrictions from certain locations	Yes 1 /No 0	
**Pricing**	***1–5***			
Tax rate calculated as percentage of price^3^		Beer	% tax	Affordability of alcohol
		Wine	% tax	
		Spirits	% tax	
**Marketing**	***1–5***			
*Legally binding restrictions on*		*For each mode*		Actual number of modes
Traditional advertising		No regulation	0	
Digital advertising		Industry self regulation	0	
Sponsorship sports/youth events		Partial ban	1	
Sales promotions		Total ban	2	
Product placement		Differential by potency	Yes 1/No 0	
**Drink driving**	***1–5***			
Blood Alcohol Content (BAC) level		No BAC	0	% vehicles stopped for Random Breath Testing
		BAC 0.05% or above	1	
		BAC between 0·03% and 0·05%	2	
		BAC between 0.0% and 0.03%	3	
Enforcement		Sobierty checkpoints	Yes 1/ No 0	
		Random breath testing/checkpoints	Yes 1/ No 0	
		Zero tolerance for professional drivers	Yes 1/ No 0	

^1^ Yes/No were scored depending on which option represented the restriction e.g. if no meant greater restriction then it was coded as 1.

^2^ Same scoring as used for legal hours.

^3^Weighted by the % of beverages consumed in each country as per WHO: Global Information System on Alcohol and Health (GISAH).

### Data sources

The Alcohol Environment Protocol (AEP) has been developed to allow countries to document and assess (in a comparable way) the policy environment in which alcohol is sold and consumed. Using the AEP, participating countries collected data on whether policies were in place, their stringency (i.e., the level of restriction), and ‘policy impact’ measures of the alcohol environment in each country.

Data on policies and their stringency were drawn from legislative documents, liquor licensing lists (hours), and websites (e.g., excise tax and BAC levels from government websites) in each country and entered into the AEP.

The data on impact measures for physical availability, tax, and marketing were obtained from specifically designed data collection (specified in the AEP). Surveys of premises were undertaken by visiting or calling common types of on- and off-premises, up to four of each, to document retail prices. Researchers also completed a schedule documenting the modes of alcohol marketing in their country. For drink driving, police data available for some high-income countries were used to assess the implementation of drink driving policy, defined as the percentage of vehicles stopped for random breath testing. These percentages were estimated for the four middle-income countries, mainly using previous research or key informant estimates combined with data on number of vehicles on the road in the country.

We obtained alcohol consumption data from the WHO. Recorded APC (15+) from GISAH [9] was used (excluding unrecorded alcohol, which is not subject to the same policy regulation as recorded alcohol). Data from the time period most comparable to the time of data collection for the IAC Policy Index was used. Per capita consumption was useful in this first stage of analysis to develop the Index as it is a good independent indicator of country differences at the individual consumption level (the distribution of alcohol consumption is very similar at different levels of per capita consumption [23]) and therefore provides a useful test dataset.

Gross domestic product for each country was obtained from World Bank national accounts data, and OECD National Accounts data files [24,25].

### The IAC policy index

The measures and effectiveness weighting parameters used for the IAC Policy Index are outlined in Table 1.

#### Stringency

The measure of policy stringency is level of policy restriction as legislated or regulated. For physical availability, restrictions on number; specified geographic area; and distance from certain locations comprised the score of outlet density for both on- and off-license. The number of hours on-premise and off premise stores were permitted by law to be open per day was documented and scored (higher score for stricter/shorter hours), and also if premises were permitted to be open for all 7 days of the week. For tax, policy stringency was calculated on tax rate. The tax rate was calculated as a percentage of price for three beverages (beer, wine, and spirits) weighted by the proportion each beverage contributed to the alcohol market. The marketing domain was made up of five sections (traditional advertising, digital advertising, product placement, sponsorship, and sales promotions). These were scored according to no regulation/industry self-regulation, a partial ban and a total ban. The drink driving policy stringency score ranged from not having a BAC limit through to a BAC between 0% and 0·03% (the strictest). Sobriety checkpoints (where suspicion of drinking is required before testing can occur), random breath testing checkpoints (where any driver can be tested without suspicion of drinking), random breath testing (where any driver can be stopped anywhere and tested), and zero tolerance for professional drivers were also included (see Table 1 for overview and see S1 Text and S1 Table for full details).

#### Impact

For the impact of *physical availability policy*, actual hours reported open were used and included in the Index using the same categories as for the legally allowed hours (Table 1). No impact measure for alcohol outlet density was available because not enough countries had or could collect these data (but this could be included in future iterations if resources are available). The affordability of alcohol in a country was assessed to gauge the impact of *tax policy*. The typical mid-price of 15ml absolute alcohol was collected in the price survey of on- and off-licenses conducted by the researchers; this was averaged over the three most common commercial beverages, weighted by the proportion each beverage contributed to the alcohol market in each country in each country (as defined by WHO data) [9], and then divided by per capita GDP to create a measure of affordability. To assess the impact of marketing policy in a country, 25 modes of marketing were measured (yes/no), and then the number of modes of marketing not present in a country divided by 25. For drink driving, the percentage of vehicles stopped for random breath testing in a country was included in the index (refer to methods section for details).

### Sensitivity analysis and selection of effectiveness weights

Different domains were initially weighted from 1 to 5 according to the effectiveness of regulations based on the available scientific evidence, with 1 being least effective and 5 being most effective [e.g., 20]. Sensitivity analysis was then undertaken to assess the robustness of the Index to changes in the effectiveness weights applied in the Index domains and to select the final weights for the Index. The analysis tested the effects of weights 1 unit higher and 1 unit lower than the initial weights, which resulted in 19,683 different combinations. The final weights selected for use in the IAC Policy Index were those providing the largest correlation with the recorded per capita consumption. Generating the final weights using this approach meant they were a combination of expert knowledge and data-driven approaches. Sensitivity analysis assessed the impact of the different weights on the ranking of countries within the IAC Policy Index (based on the correlations at country-level) and the effect of leaving each country out of the Index.

The sensitivity analysis generally provided confidence that the ranking of countries and outcomes of the IAC Policy Index were not dependent on changes in the weights of the domains. In all cases, the country rank when baseline weights were applied was also the most common rank when the sensitivity analysis weighting iterations were applied.

The correlations with recorded APC in a country were quite consistent throughout all iterations. The interquartile range was 0·04 (-0.85 to -0.81) The effect of leaving each country out, one at a time, on the correlations with recorded APC was minimal.

### Missing data

Alcohol outlet density (an impact measure for physical availability) was missing for most middle-income countries and therefore could not be included in the Index. Data were not available for the percentage of vehicles stopped for random breath testing in one country and so the percentage was imputed (see analysis section). Four other countries estimated this percentage based on previous research or key informant estimates and data on the number of cars on the road in a country.

### Analysis

The IAC Policy Index generated scores with a potential range of 0 to 25 points. We then used the Index to assign a score to each country: in each domain, data collected for the Index were converted into a score between zero and one, with a higher score representing more stringent policy and evidence of more restrictive on-the-ground impact. Some of the data collected had to be inversed so that this direction was maintained. Once standardised, values in each domain were then weighted by between one and five to reflect effectiveness and then summed to make up the total IAC Policy Index score for each country.

A cross-sectional analysis of the 12 countries was conducted. The scores (for each policy domain separately and for the overall IAC Policy Index) were correlated with Recorded APC. The association between APC and the tax design and price/tax ratio was also examined separately.

The analysis was undertaken using Excel and R Version 4.1.

## Results

The countries were ranked using the IAC Policy Index. The score for each domain and the total scores are shown in Table 2.

**Table 2:** Countries ranked by IAC Policy Index and domain scores; higher scores indicate more restrictive policy).

	Rank	Hours	Outlet density	Drink driving	Pricing	Marketing	Total
Turkey	1	1.1	1.0	2.6	2.3	7.0	13.9
Vietnam	2	0.6	2.0	1.7	6.1	1.4	11.8
Thailand	3	1.3	1.0	3.0	2.2	2.8	10.3
Mongolia	4	0.7	1.0	2.1	3.2	2.7	9.7
St Kitts and Nevis	5	1.5	2.0	0.9	1.5	2.2	8.0
Chile	6	1.4	0.5	3.2	1.9	0.7	7.6
South Africa	7	1.0	1.0	2.3	1.5	0.9	6.7
Scotland	8	1.1	1.5	0.4	1.9	1.6	6.5
Australia	9	0.6	0.0	2.6	1.2	1.4	5.8
England	10	0.7	0.0	0.9	2.3	1.3	5.1
Netherlands	11	0.7	0.5	1.8	1.1	1.0	5.0
New Zealand	12	0.4	0.0	1.8	1.5	1.4	5.0

Cross-sectional correlations with recorded APC are shown separately for each domain for policy stringency, impact, and combined policy stringency plus impact (the IAC Policy Index) (see Table 3).

**Table 3:** Correlation with recorded APC (litres of pure alcohol).

	Policy Stringency	Impact	IAC Policy Index
Pricing	-0.02	-0.80	-0.64
Hours and days of sale	-0.12	0.23	0.00
Outlet density	-0.44	Not available	-0.44
Marketing	-0.57	-0.56	-0.68
Drink driving	-0.45	0.27	-0.34
Total	-0.76	-0.65	-0.91

**Fig 1:**
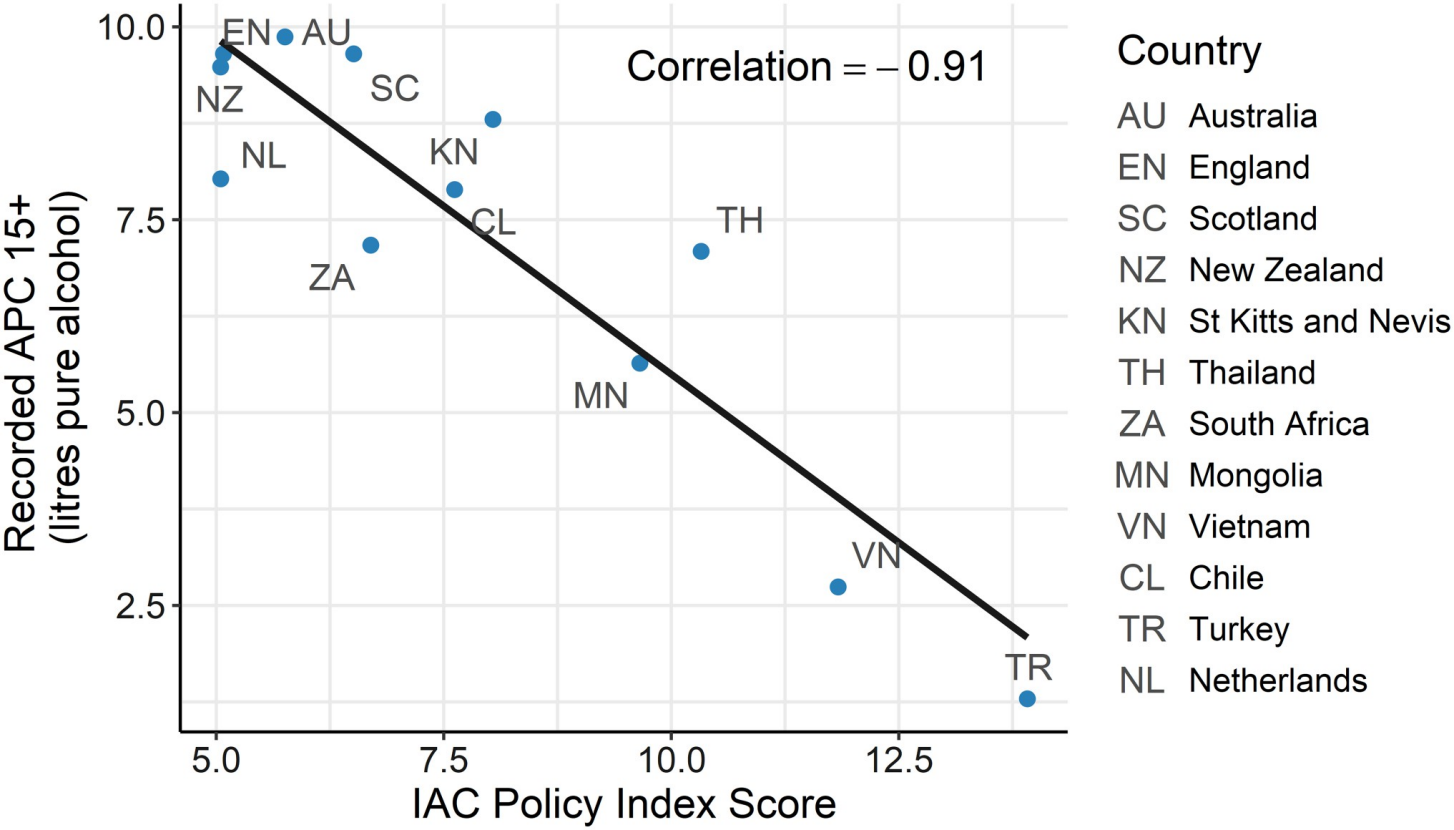
Relationship of policy index with recorded APC.

The overall correlation between the IAC Policy Index and recorded APC was -0.91 (Fig 1). Table 3 shows the correlations for the different domains by stringency and impact.

For all domains a negative correlation signifies that more stringent policy and more restrictive on-the-ground impact is associated with lower recorded alcohol.

**Fig 2:**
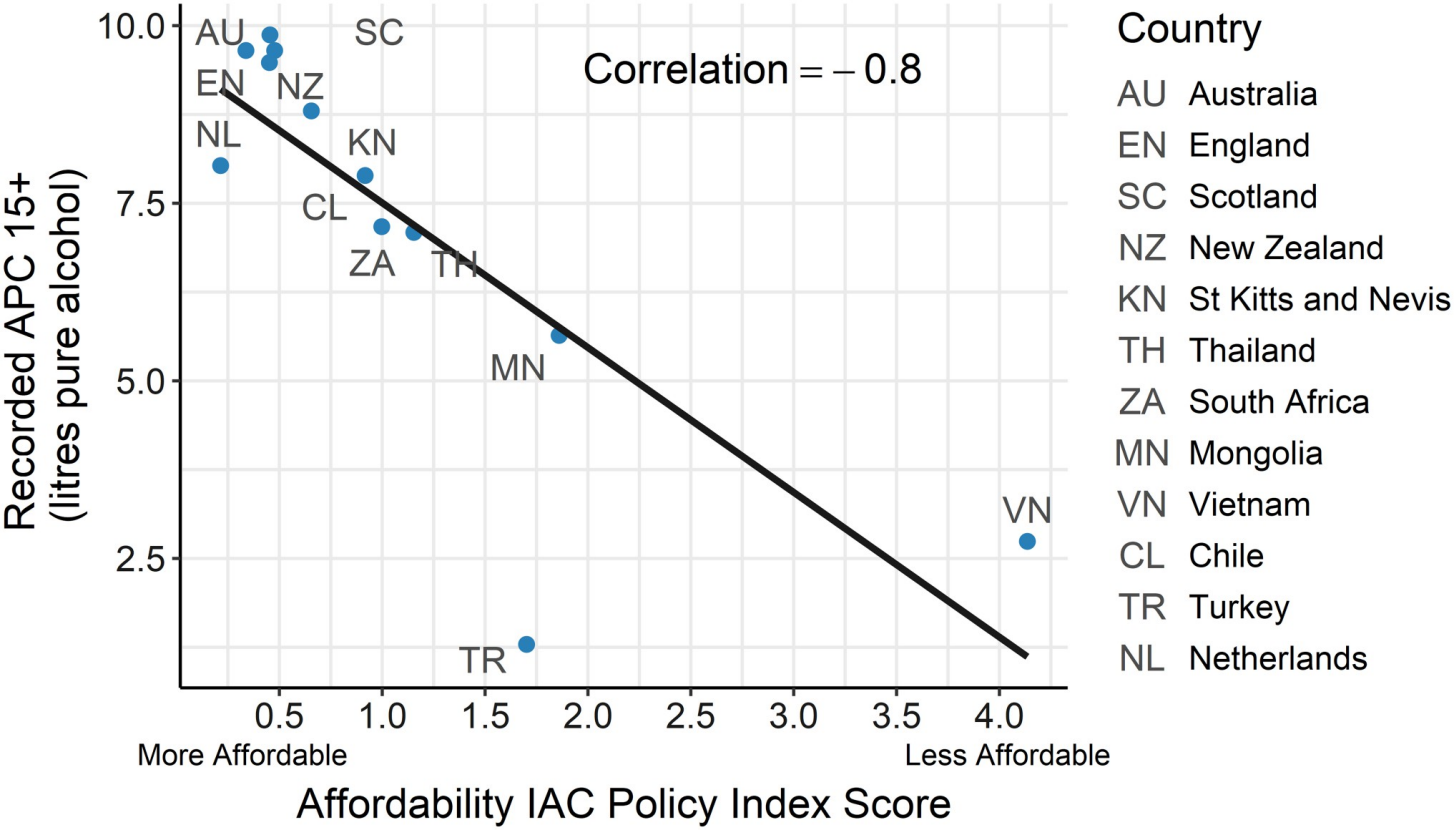
Relationship of affordability of commercial alcohol with recorded APC.

Tax policy stringency was correlated positively with APC and the separate analyses of the two components found this reflected a positive 0.57 correlation between APC and the tax design scores assigned. There was no correlation (-0.02) found with price/tax ratio. In contrast, affordability, the measure of on-the-ground pricing impact, was highly negatively correlated with APC (Fig 2).

**Fig 3:**
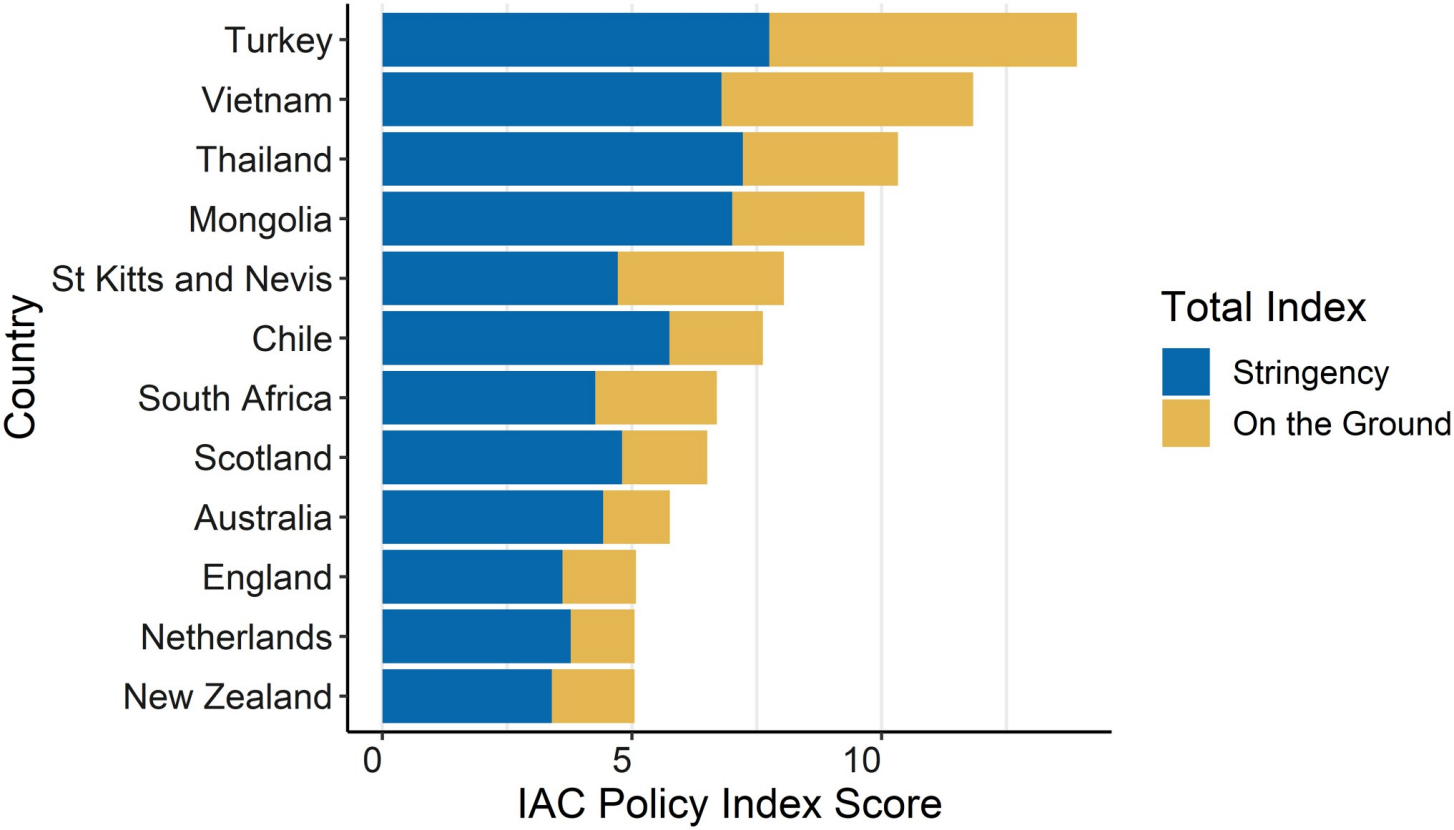
The contribution made by stringency and on-the-ground impact for each country.

The dispersion of the countries in relation to the IAC Policy Index showed a general pattern of higher income countries with fewer policy restrictions, less restrictive on-the-ground conditions, and higher consumption. The measures of on-the-ground impact were highly correlated (0.70) with the policy stringency when analysed across countries (Fig 3).

## Discussion

### Key findings

The IAC Policy Index created in this study correlated negatively and highly with recorded APC across a diverse group of countries. Alcohol per capita consumption, a reliable indicator of alcohol use and for which there is annual data available in most countries of the world [26], is commonly used to assess the usefulness of policy indices. The relationship found in this study using the finalised IAC Policy Index was stronger than in previous analyses. Several of these analyses have used a number of additional policy and infrastructure measures (14) and taken into account country characteristics such as GDP (12). The strength of the relationship suggests the IAC Policy Index’s focus on a small number of key policies, as well as the decision to collect data on not only stringency of legislation but also the alcohol environment impact measures, have resulted in a useful policy index. Apart from the lack of data on alcohol outlet density, there was very little missing data and researchers in-country were able to complete the systematic tool (the Alcohol Environment Protocol).

### Study innovation and implications

An important innovation of this study was the inclusion of a measure of the impact of alcohol policies. The impact measures are a reflection of the real-world alcohol environment the policies are intended to affect and should reflect both policy and implementation/enforcement (although they are also affected by other factors). These impact measures were found to be strongly associated with APC, and more strongly associated with APC in some domains than the measures of policy stringency. The addition of the impact measures also improved the IAC Policy Index’s overall correlation with recorded APC.

The biggest difference between policy stringency and on-the-ground impact measures was found for pricing. The measure we derived for policy stringency of taxation, the tax/price ratio, an indicator widely used in tobacco control, showed a small negative correlation with APC, while the negative correlation with the impact measure of affordability was very strong. There is increasing evidence that affordability, which reflects price and income, is the impact measure that taxation policy needs to affect. In this study affordability, prices adjusted by GDP, was highly negatively correlated with APC. Affordability affects consumption [27] and all-cause mortality [28] and beer has been increasing in affordability globally [29]. The price/tax ratio has been critiqued in the context of tobacco policy in LMICs in which the economy is growing and thus affordability has proved to be a more appropriate measure [30]. These findings suggest alcohol tax rates need to influence affordability and the measures used to monitor them need to reflect this. Measures of taxation policy that do not have these measures available are less likely to be relevant [9].

Alcohol marketing was found to be an important policy domain in terms of impact. The impact measure of marketing was closely related to the stringency measures, suggesting that whatever marketing approaches were legal were employed in that country. This finding highlights the importance of having alcohol marketing policy restrictions in place in countries. The time period over which most of these data were collected predated the major expansion of digital marketing of alcohol. While data were collected on digital marketing, this could be expanded in future studies.

### Other policy domains

One measure of availability included in the IAC Policy Index was hours and days of sale. This showed no association with APC. The research literature showing an effect of curtailing or extending hours of sale is largely based on harm measures, particularly violence associated with late night trading of on-premises [31–33]. One study of a change in trading hours showed an effect on harm, but not consumption [34]. It is therefore possible that hours of sale have an effect on alcohol harms, which does not, however, affect aggregate consumption.

Unfortunately impact measures relating to another measure of physical availability, outlet density and spatial positioning, are not readily available in many LMICs, although a protocol for an observational study to collect an indicator measure developed in Thailand is available as part of the IAC study [19]. The stringency measure alone showed the expected relationship with APC, but future development of the IAC Policy Index could investigate the use of data from observational studies where appropriate.

Drink-driving policy was included in the Index since this policy is of considerable importance due to the contribution of drink driving to alcohol-related trauma. It was hypothesised drink driving policy would not have a high association with APC as it is primarily focused on reducing road traffic trauma, not consumption. However, the stringency of drink driving measures did show a relationship in the expected direction. The impact measure, the percentage of vehicles stopped for random breath testing, however, did not. High-income countries have more resources to enforce drink driving policy, which likely resulted in higher percentages of drivers being tested. This combined with higher recorded per capita relative to the middle-income countries may explain the result.

### Limitations

This is a cross sectional study and the associations between policy status and APC are likely to have causal relationships in both directions. Based on one year of APC data we cannot know the underlying trend. The APC trend could affect the interpretation of the accuracy of the IAC Policy Index. Longitudinal analysis using this tool will give a clearer understanding of these relationships.

Lack of data is a limitation. The lack of impact measures for outlet density and spatial positioning needs to be taken into account when interpreting results. For example, Vietnam scores highly on stringency in this area as it has strong regulation in place. However, there is a lack of enforcement and alcohol is widely sold [35]. The data on the extent of random breath testing was estimated in four countries mainly using previous research, or key informant estimates combined with data on number of vehicles on the road in the country.

The LMICs in this convenience sample included some countries with relatively strong policies in place as this convenience sample relied on availability and collaboration with alcohol researcher colleagues in those countries. Thailand, for example, has been identified as having slowed the expected increase in alcohol consumption given its economic growth through the application of comprehensive alcohol policies [36]. The current finding that LMICs had more stringent policy and impact measures compared with the HICs may not be true of a broader sample of countries.

## Conclusions

The IAC Policy Index provides a valuable tool to assess alcohol policy developments over time and between countries. This study demonstrates the value of incorporating the impacts of policies, reflecting the implementation of policies, as well as policy stringency, in policy indices. The implication from this study for refining measures of pricing policy is especially important given the empirical evidence of its effectiveness for reducing alcohol harm.

## Supporting information

S1 Text Calculation of IAC alcohol policy index. (DOCX)

S1 Table Final effectiveness weights. (DOCX)
