# Correction: Benchmarking alcohol policy based on stringency and impact: The International Alcohol Control (IAC) Policy Index

**DOI:** 10.1371/journal.pgph.0000592

**Published:** 2022-05-31

**Authors:** The PLOS Global Public Health Staff

## Notice of republication

This article was republished on May 17, 2022, to correct the XML version of the article which displayed an incorrect notation beside two authors names in the byline. The publisher apologizes for the errors. Please download this article again to view the correct version. The originally published, uncorrected article and the republished, corrected articles are provided here for reference.

## Supporting information

S1 File Originally published, uncorrected article. (PDF)

S2 File Republished, corrected article. (PDF)
