# Supplementary material for: Benchmarking alcohol policy based on stringency and impact: The International Alcohol Control (IAC) policy index
Source: PLOS Glob Public Health. 2022 Apr 22;2(4):e0000109. doi: 10.1371/journal.pgph.0000109 (PMC10021514; doi:10.1371/journal.pgph.0000109)
Supplement: S1 Text — (DOCX) [file pgph.0000109.s001.docx]

**Calculation of IAC Alcohol Policy Index**

**Stringency**

**Physical availability stringency**

For alcohol outlet density, restrictions on number; specified geographic area; and distance from certain locations comprised the score for outlet density for both on- and off-license. These each received a score of 1 if restrictions were in place.

The number of hours on-premise and off premise stores were permitted in law to be open per day was converted to a score between 0 and 1. Zero was given to countries that had alcohol premises open for 24 hours and 1 to countries that had the shortest hours (hours shorter than 8). An additional score of 1 was given if a premise was *not* permitted to be open for all 7 days of the week.

**Tax stringency**

Tax rate was calculated as a percentage of retail price for three beverages (beer, wine, and spirits) weighted by the proportion each beverage contributed to the alcohol market. Where tax design differed by beverage an overall score was calculated proportional to the consumption of the different beverages.

Minimum unit pricing was included in the index but was not in place in any jurisdictions at the time of the study and as such contributed no score.

**Marketing stringency**

The marketing domain was made up of five sections (traditional advertising, digital advertising, product placement, sponsorship, and sales promotions) and difference by potency. No regulation/, Industry self-regulation was scored 0, a partial ban scored 1, while a total ban scored 2. If restrictions did not differ by potency of the beverage (e.g. more liberal for lower potency beverages), then an additional score was assigned. The scores were averaged and summed across media.

**Drink driving stringency**

A BAC level of 0·05% or above was scored 1, between 0·03% and 0·05% was scored 2, and a BAC level between 0% and 0·03% was scored 3.

Sobriety checkpoints (where suspicion of drinking is required before testing can occur), random breath testing checkpoints, random breath testing (where any driver can be stopped anywhere and tested), and zero tolerance for professional drivers all scored 1 if in place and 0 if not.

**Impact measures**

**Physical availability impact measure: Actual hours of trading**

Alcohol outlet density was excluded as data were not available for most countries

Reported actual hours open used the same method as for the legally allowed hours. The number of hours on-premise and off premise stores were permitted in law to be open per day was converted to a score between 0 and 1. Zero was given to countries that had alcohol premises open for 24 hours and 1 to countries that had hours shorter than 8. An additional score of 1 was given if a premise was *not* permitted to be open for all 7 days of the week.

### Tax Impact measure: Affordability of alcohol

The typical mid-price of 15ml absolute alcohol, collected by surveying prices on- and off-licenses, was averaged over three commercial beverages and was weighted by the proportion each beverage contributed to the alcohol market. This was divided by per capita GDP to create a measure of affordability.

**Marketing impact measure: Actual number of modes**

The presence/absence of twenty-five types of marketing was asked about in each country. The sum of the absent of types of marketing for a country was divided by the total score possible (25).

**Drink driving impact measure: % vehicles stopped for random breath testing.**

The impact measure for drink driving was the percentage of motorised vehicles stopped for random breath testing in a country. Four countries estimated this percentage mainly using available research, data and key informant estimates. One country had missing data (Chile) and this value was imputed using the average across all countries.
