# Supplementary material for: Benchmarking alcohol policy based on stringency and impact: The International Alcohol Control (IAC) policy index
Source: PLOS Glob Public Health. 2022 Apr 22;2(4):e0000109. doi: 10.1371/journal.pgph.0000109 (PMC10021514; doi:10.1371/journal.pgph.0000109)
Supplement: S1 Table — (DOCX) [file pgph.0000109.s002.docx]

**S 1Table. Final Effectiveness weights**

| **Stringency** | **Weights** |
| --- | --- |
| Hours and days of sale | 1 |
| Outlet density | 3 |
| Drink driving | 3 |
| Pricing | 4 |
| Marketing | 3 |
| **On the ground Impact** |  |
| Hours and days of sale | 1 |
| Drink driving | 1 |
| Price | 5 |
| Marketing | 4 |
